# Attitudes towards COVID-19 vaccination: A cross sectional study in the Federal Capital Territory, Nigeria

**DOI:** 10.1371/journal.pgph.0002589

**Published:** 2024-04-18

**Authors:** Obi Peter Adigwe, Godspower Onavbavba

**Affiliations:** National Institute for Pharmaceutical Research and Development, Abuja, Nigeria; University of Michigan, UNITED STATES

## Abstract

Vaccine acceptance is complex and context-specific, varying across time, place and perceived behavioural nature of the community involved. A high vaccine acceptance rate is important to achieve herd immunity, however, vaccine hesitancy is a possible barrier to this. This study aimed to assess attitudes towards COVID-19 vaccination and associated factors. A cross-sectional survey was undertaken to investigate the attitudes of Federal Capital Territory residents towards COVID-19 vaccine uptake in Nigeria. Data were collected using questionnaires which were administered to respondents physically and online through random and snowball sampling strategies respectively. Data received were then analysed using Statistical Package for Social Sciences (SPSS). A total of 1767 responses were received with males representing 57.8% of the sample. More than half (54.9%) of the respondents were between the ages of 18–30 years. A third (35.4%) of the study participants indicated that a vaccine was not necessary for COVID-19, and 56.5% indicated willingness to accept COVID-19 vaccination. The majority of the sample (56.9%) indicated that the government decision-making was in their best interest, whilst close to two-thirds of the respondents (61%) were of the view that COVID-19 vaccination should not be made compulsory. Older respondents as represented by those over 60 years were more likely to accept COVID-19 vaccination (*p* = 0.039). This study however revealed negative attitudes towards COVID-19 vaccination, indicating the urgent need for government, policymakers, and other stakeholders to prioritise the development of strategies that can appropriately address vaccine hesitancy in the study setting. Contextual interventions indicated include strategic public enlightenment campaigns targeting populations with less favourable dispositions towards being vaccinated.

## Introduction

COVID-19 emerged in December 2019 and has since then been associated with an increasing rate of infections and deaths globally [1]. The disease is caused by SARS-CoV-2 which belongs to the class of coronavirus. It was first identified in Wuhan [2], and later spread to different countries before being declared a pandemic by the World Health Organization [3, 4]. In Nigeria, the first case of COVID-19 was announced on the 27^th^ of February 2020, when an Italian citizen came into the Country and tested positive for the disease [5]. The outbreak in Nigeria led to significant disruptions in daily life and economic activities in the Country.

Prior to the COVID-19 pandemic, research activities to develop a vaccine against severe acute respiratory syndrome (SARS) and Middle East respiratory syndrome (MERS) had established knowledge about the structure of coronaviruses [6]. This, alongside emergent innovations, accelerated various relevant development processes with respect to vaccines’ technology for COVID-19. By mid-December 2020, several COVID-19 vaccines were in clinical trial stages and many of them demonstrated efficacy as high as 95%, in preventing the disease. National drug regulatory authorities of several countries gave approval for some of these vaccines for public use, and this prompted the commencement of vaccination in a number of nations [7].

In March 2021, Nigeria obtained an estimated 4 million doses of the AstraZeneca/Oxford vaccine as part of the global efforts to address the COVID-19 pandemic. Despite the Government’s effort to ensure that its citizens get vaccinated against the disease, several factors affected the vaccine acceptance rate [8]. These include distrust in the public interventions, conspiracy theories, misinformation, and fear of adverse effects [9]. Pain at the injection site, fatigue and headaches were the major side effects reported amongst individuals who received the AstraZeneca/Oxford vaccine in Nigeria [10]. These challenges corroborated the vaccine uptake theory which explains vaccine acceptance amongst individuals as being influenced by the multidimensional construct of vaccine confidence, vaccine hesitancy, and vaccine access [11]. Understanding the vaccine uptake theory can help public health actors in the development of strategies to increase vaccine uptake and reduce hesitancy.

Vaccine hesitancy refers to reluctance or refusal to be vaccinated or to have one’s children vaccinated against contagious diseases [12]. It was identified by the World Health Organisation as one of the top ten global health threats of 2019 [13, 14]. The term encompasses outright refusal to vaccinate, delaying vaccination, accepting vaccines but remaining uncertain about their use, or using certain vaccines and refusing others [15, 16]. Arguments against vaccination are however contradicted by overwhelming scientific consensus about the safety and efficacy of vaccines [17–19].

Vaccine hesitancy stems from multiple factors including lack of confidence and issues associated with complacency and convenience [20]. Lack of confidence can arise as a result of mistrust of vaccines or healthcare providers, whilst complacency may come up when hesitant individuals do not see the need or value of vaccines [15]. Vaccine hesitancy has been in existence since the invention of vaccines, and this phenomenon often results in disease outbreaks and deaths from vaccine-preventable diseases [21–24]. In many countries, vaccine hesitancy and misinformation present substantial obstacles to achieving herd immunity [25]. The emergence of COVID-19 re-ignited the debate about the acceptance of vaccines across different parts of the world, with some opining that support for vaccines development may be underpinned by ulterior motives, rather than the prevention of the disease [26]. Some studies had explored the acceptance of COVID-19 vaccines prior to their development [27–29]. Misinformation, safety, and novelty of the vaccines were among the most important deterrents to vaccination in these studies. In line with these factors, about 20% of the respondents in a survey carried out amongst US citizens indicated that they would decline COVID-19 vaccination, and more than a quarter of participants in a study undertaken in China expressed hesitancy, in relation to the vaccine under consideration [27, 28]. Vaccine acceptance is however reported to be context-specific and varies with geography, culture, and demography [30]. It is against this backdrop that this study aimed at assessing attitudes towards COVID-19 vaccination and associated factors in Federal Capital Territory of Nigeria.

## Materials and methods

### Study design and setting

A cross-sectional survey was undertaken in Nigeria between July 20, 2022 and December 15, 2022, to gain an understanding of the attitudes of residents towards COVID-19 vaccination [31]. The research was carried out in the Federal Capital Territory which was reported to have recorded the highest cases of COVID-19 in Nigeria, after Lagos state [32]. Nigeria has the largest population in Africa and recorded the eleventh highest COVID-19 cases in the Continent [33]. Since the COVID-19 pandemic, there have been *267*,*153* confirmed cases of COVID-19 with 3,155 deaths, reported to WHO as of December 29, 2023 [34].

### Study population

The study included individuals who were residents of the Federal Capital Territory of Nigeria and were 18 years or older. Participants who did not meet these criteria were excluded from the study. The study population also comprised both male and female participants with varying levels of education and a wide range of occupations.

### Data collection tool

The questionnaire (S1 File) for the study was developed following a review of existing literature [27–29]. The questionnaire items were developed with input from researchers with relevant experience in this area. The research instrument was structured to gain insight into the attitudes of the population towards COVID-19 vaccines. The questionnaire comprised two sections, the first which aimed at collecting demographic details, whilst the second explored attitudes towards COVID-19 vaccines. The socio-demographic characteristics section was made up of five questions, whilst the attitudes section had a total of seven questions. A four-point Likert scale was used for the items exploring respondent’s attitudes, including strongly disagree, somewhat disagree, somewhat agree, and strongly agree.

Face and content validations of the instrument was undertaken using an independent expert panel comprising five researchers with robust experience in the area of public health research. Face validity involved assessing the research instrument for appropriateness, complexity, attractiveness and relevance. A draft version of all the items in the instrument was reviewed independently by each expert, and they suggested changes, additions, and deletions. The revision process continued until a consensus was reached. Content validity was carried out using a quantitative method. The tests carried out include content validity ratio and content validity index, and only items that passed these tests were included in the final questionnaire. Also, the Cronbach alpha test was undertaken for the instrument, which gave value of 0.712, thereby indicating internal consistency among the questionnaire items. The questionnaire was tested in a pilot phase by physically administering it to a cohort of 21 participants that were randomly selected. The feedback received did not result to any major changes. These 21 questionnaires were therefore included in the final analysis.

### Sampling and data collection

Data were collected using both online and physical methods so as to get a good number of participants in the study. Using the Epi Info software version 7, a minimum sample size of 1536 was calculated for a population of approximately 3.8 million residents in the Federal Capital Territory at 95% confidence level, 2.5% margin of error, and 50% response distribution [35]. The participants were sampled using both snowball and random techniques. The snowball sampling method was adopted for online data collection so as to increase the number of participants [36, 37]. Online questionnaire was sent to various WhatsApp groups and Facebook pages comprising residents of the Federal Capital Territory. The questionnaire was posted on these platforms and participants who clicked on the link were directed to a Google form to indicate their responses. Participants were asked to click the submit option after completing the questionnaire. The respondents were also directed to share the link to the questionnaire with their friends, family, colleagues, and associates residing in the Federal Capital Territory. Those who indicated that they were non-residents of the Federal Capital Territory were excluded. Hard copies of the questionnaire were also administered using a random sampling strategy [38]. This was to ensure that participants who lacked access to the internet were included in the study. The respondents were randomly selected from the six area councils in the Federal Capital Territory. Locations such as worship centres, motor parks, and corporate offices are examples of strategic sites selected to ensure data collection democratisation.

### Ethics consideration

Prior to the collection of data, ethical approval was obtained from the National Institute for Pharmaceutical Research and Development Health Research Ethics Committee with approval number: NIPRD-HREC/039/21A. Participation in this study was voluntary and written informed consent was obtained from participants. Confidentiality was appropriately maintained by not including the names of the study participants in the questionnaire.

### Data analysis

Data retrieved from the survey were prepared in Microsoft Excel format and rechecked for accuracy. The data were then imported into Statistical Package for Social Sciences (SPSS) version 25 for analysis and were also encrypted for safety. Descriptive statistical analysis was undertaken, and a chi-square test was used to determine the association between responses and socio-demographic characteristics of the study participants [39]. Prior to carrying out the chi square test, the responses of the participants were aggregated to have just two responses which include “disagree” and “agree”. A *p-*value of less than 0.05 represented the threshold for statistical significance.

## Results

### Socio-demographic data

A total of 1767 responses were received from the data collection process which comprised 1446 paper-based responses and 321 online responses. The response rate for physical administration was 85.06%. Male participants were in the majority as indicated by 57.8% of the sample, more than half (54.9%) of the respondents were between the ages of 18 to 30 years, whilst those above 60 years represented the least proportion of the sample (3.2%). In the area of education, those with first degrees represented more than half (53.8%) of the study participants surveyed, whilst those with primary school education represented the least (2.5%). Occupationally, a third (33.6%) of the study participants were engaged in the private sector. Further details about socio-demographic characteristics are presented in Table 1.

**Table 1:** Socio-demographic characteristics.

Variables	Frequency (%)
Gender	
Male	1022 (57.8)
Female	745 (42.2)
Age (years)	
18–30	967 (54.9)
31–40	428 (24.2)
41–50	239 (13.5)
51–60	73 (4.1)
Above 60	57 (3.2)
Education	
Primary	45 (2.5)
Secondary	227 (12.9)
National diploma/NCE	260 (14.7)
First degree/HND	950 (53.8)
Postgraduate	284 (16.1)
Occupation	
Unemployed	235 (13.3)
Self-employed	386 (21.8)
Private	594 (33.6)
Government sector	454 (25.7)
Retired	44 (2.5)
Others (e.g. Students)	54 (3.1)

### Attitudes towards COVID-19 vaccination

A total number of 1742 participants representing 98.6% of the sample indicated that they had never been infected with COVID-19. More than half (52.9%) of the respondents indicated that they would accept vaccination against COVID-19 as a prerequisite for employment, whilst 47.1% indicated that they would not accept COVID-19 vaccination if it was part of the criteria for employment.

A third (35.4%) of the study participants indicated that the chance of getting COVID-19 is so low that vaccination was not necessary, and 43.3% indicated that they would not accept the COVID-19 vaccine. Only 56.7% of the study participants indicated that the Government was making decisions in their best interest. Further details relating to attitudes towards COVID-19 vaccination are presented in Table 2.

**Table 2:** Attitude towards COVID-19 vaccination.

	Statement	Strongly Agree	Somewhat Agree	Somewhat Disagree	Strongly Disagree
1	The chance of getting COVID-19 is so low that a vaccine is not necessary.	292 (16.6)	332 (18.8)	476 (26.9)	660 (37.5)
2	I would accept to be vaccinated against COVID-19.	425 (24.1)	573 (32.4)	346 (19.6)	419 (23.7)
3	Government is making decisions in your best interest with respect to vaccination.	304 (17.3)	698 (39.6)	445 (25.3)	314 (17.8)
4	COVID-19 vaccination should be made compulsory for every citizen.	337 (19.1)	351 (19.9)	507 (28.8)	567 (32.2)
5	Pharmaceutical companies have your best health interest for developing COVID-19 vaccine.	475 (27.0)	724 (41.1)	365 (20.7)	198 (11.2)
6	Non-governmental entities supporting COVID-19 vaccine research have your best interest.	378 (21.4)	878 (49.8)	367 (20.8)	140 (7.9)

Following the descriptive statistical analysis undertaken on the data, cross-tabulation was undertaken to further interrogate associations with the responses of the study participants. Findings from this analysis revealed that some of the responses of the study participants were influenced by their socio-demographic characteristics. In Table 3, a strong majority (80%) of those who had been previously infected with COVID-19 indicated acceptance of COVID-19 vaccination as compared to only 56.3% of those never been infected with the disease. This finding was statistically significant (*p* = 0.017). Also, the majority of participants who were above 60 years of age were more likely to be vaccinated against COVID-19 as 71.9% indicated acceptance compared to other age groups which varies from 55.1% to 62.3% (*p* = 0.039).

**Table 3:** Cross-tabulation of some variables with willingness to be vaccinated against COVID-19.

Variables	Agree (%)	Disagree (%)	X^2^	*p-value*
**Ever been infected with COVID-19**				
Yes	20(80.0)	5 (20.0)	5.649	0.017
No	978 (56.3)	760 (43.7)		
**Gender**				
Male	592(58.1)	427(41.9)	2.177	0.140
Female	406(54.6)	338(45.4)		
**Age**				
18–30	532 (55.1)	434 (44.9)	10.101	0.039
31–40	235 (55.0)	192 (45.0)		
41–50	149 (62.3)	90 (37.7)		
51–60	40 (54.8)	33 (45.2)		
Above 60	41 (71.9)	16 (28.1)		
**Highest Educational Level**				
Primary School	28(62.2)	17(37.8)	27.980	<0.001
Secondary School	151(66.5)	76(33.5)		
National Diploma/NCE	161(61.9)	99(38.1)		
First Degree/HND	483(50.9)	465(49.1)		
Postgraduate	174(61.7)	108(38.3)		
**Occupation**				
Unemployed	121(51.5)	114(48.5)	16.760	0.005
Self-Employed	204(53.0)	181(47.0)		
Private Sector	342(57.8)	250(42.2)		
Government Sector	259(57.2)	194(42.8)		
Retired	34(77.3)	10(22.7)		
Other	38(70.4)	16(29.6)		

## Discussion

Several interesting findings emerged from this study which are critical for government, policymakers, and public health experts, in their decision-making regarding COVID-19 immunisation. A third of the study participants had indicated that COVID-19 vaccine was not necessary, suggesting not only their lack of interest in getting vaccinated against the disease but also a propensity towards discouraging others. Close to half of the study participants were not willing to get vaccinated against COVID-19. This finding suggests a high rate of COVID-19 vaccine hesitancy among the population. The implication of this is that it may be difficult to achieve herd immunity which is important to control contagious diseases in the community [40]. This poor vaccine acceptance rate can also be attributed to amongst other things, the false information and rumours that the COVID-19 vaccine affects fertility [41]. Available evidence indicates that only a quarter of the entire Nigerian population that are eligible for COVID-19 vaccination have been duly vaccinated, more than two years following the roll-out of the intervention [42]. Herd immunity, which is an indirect form of protection from contagious disease can only be achieved when a sufficient percentage of a population has become immune to an infection. The percentage of the population who need to be immune to achieve herd immunity varies with each disease, for instance, herd immunity against measles requires about 95% of the population to be vaccinated [43]. However, report suggests that more than three quarters of the population need to be vaccinated so as to achieve herd immunity for COVID-19 [44]. Herd immunity for infectious disease can be achieved through vaccination or previous infection and can help reduce the likelihood of infection for individuals who lack immunity [40]. Available evidence suggests that immune individuals can help break the chain of disease transmission, thereby leading to a reduction or complete disruption in the spread of infectious disease and the consequent rates of mortality [45]. This is so because the greater the number of immune individuals in a community, the smaller the probability that non-immune persons will come in contact with an infectious individual [46]. Once herd immunity threshold has been achieved, contagious disease gradually disappears from the community and individuals who are not immune would be protected from the disease [47]. Whilst herd immunity can also be achieved by allowing the spread of the disease due to the poor vaccination uptake, this must however be discouraged as it can lead to unnecessary cases of death, especially amongst vulnerable individuals or persons with underlying health conditions [48]. It is therefore important for government, policymakers, and other relevant stakeholders to develop contextual strategies that can help increase COVID-19 vaccine acceptance among Nigerians.

A cross-tabulation of socio-demographic data with vaccine acceptance revealed that individuals with higher educational levels were less willing to get vaccinated and this may be attributed to the type of information received by this category of participants. The hesitancy demonstrated towards COVID-19 vaccination by the participants can be linked to misinformation being spread about COVID-19 vaccination [49]. Evidence exists, which links limited information as regards risk-benefit ratio of vaccination, with the belief of conspiracy theories against COVID-19 [8]. Across different countries, political actions against COVID-19 misinformation were implemented. For instance, in the United States, initiatives such as the COVID-19 Corps which involved a network of local voices and trusted community leaders to encourage vaccination were implemented [50]. Partnerships with social media platforms to amplify authoritative information from public health experts were also carried out [51]. Emergent findings from this study support similar initiatives involving policy actors, health authorities and media outlets in the provision of clear, consistent, and scientifically verified messages about vaccines, as a means of communicating proven benefits of vaccination in Nigeria and similar study settings.

A cross-tabulation of age with acceptance revealed that older people who were above 60 years were more likely to accept COVID-19 vaccination as close to a quarter of participants in this age group indicated acceptance of a vaccine. The reason behind this may be due to findings suggesting that older people were more likely to suffer severe COVID-19 complications and consequently die from the disease [52–54]. This finding is also similar to findings from a study undertaken in Saudi Arabia [30]. Furthermore, participants who have had COVID-19 previously were more likely to accept a vaccine for the disease. Although the proportion of previously infected individuals was relatively few in the sample, this novel insight provides new knowledge as regards the perspectives of this population group. Further studies can be undertaken to target a larger sample of this specific population.

In this study, close to half of the study participants disagreed that government decision-making with respect to vaccination was in their best interest. Given the level of vaccine hesitancy reflected from this study, it can be inferred that authority distrust of population, interferes with COVID-19 vaccine acceptance. Mistrust of the government by the citizens is not a new phenomenon in Nigeria. World Economic Forum (2018) ranked Nigeria among the top seven countries with the highest level of citizen distrust of government officials [55]. Literature has also revealed that political distrust interferes with peoples’ cooperation during public health interventions, thereby promoting the spread of viral diseases [56, 57]. This suggests a likelihood of contravening government guidelines regarding the prevention and control of COVID-19. Building the citizens’ confidence in government decision-making is a critical factor that can promote acceptance of COVID-19 vaccine as well as other public health interventions. The majority of the study participants however disagreed that COVID-19 vaccination should be made compulsory for all citizens. Also, close to half of the study participants indicated that they would not accept the COVID-19 vaccine if their employer insisted on them getting vaccinated before being employed. This proportion was slightly higher than those who initially indicated non-acceptance of the vaccine. This novel finding from our study differs from a previous report on global acceptance of the COVID-19 vaccine where a majority of the participants indicated that they would accept COVID-19 vaccine if employers recommended it [29]. For policymakers, employers of labour and other relevant stakeholders, it is therefore important to collectively focus on strategies to increase vaccine acceptance among the citizens, rather than making it compulsory.

About a third of the study participants disagreed with the notion that altruism underpinned pharmaceutical companies’ motivation for developing the COVID-19 vaccine. This could perhaps be attributed to a series of campaigns by anti-vaccination groups, claiming that pharmaceutical companies were trying to profit from the COVID-19 pandemic by developing vaccines [58]. Also, close to a third of the study participants disagreed that non-governmental entities supporting COVID-19 vaccine research have their best interest. Previous findings were similarly sceptical of the overarching objectives of pharmaceutical industries and non-governmental organisations, regarding COVID-19 vaccines [59]. Trust in government, pharmaceutical industries and health interventions informs decision making to participate in COVID-19 vaccination. The dynamics however varies across different settings. For instance, previous work in Denmark, USA and Ghana which identified contributory factors such as miscommunication, bureaucratic challenges, and historical pandemics unpreparedness [60]. Further research can help enable a better understanding of this phenomenon within the Nigerian landscape. Contextual reforms underpinned by these emergent findings can improve confidence not just for COVID-19 vaccination, but also for other government interventions associated with sustainable access to vaccines and other pharmaceuticals. This is therefore a critical step towards achieving Medicines’ Security alongside the consequent health access improvement, and attainment of Universal Health Coverage [61, 62].

Although the study was carefully designed to match the research objectives, there may be limitations with the sampling approach adopted. The snowball sampling strategy employed for online participants has been associated with certain forms of bias. Steps however taken to mitigate this weakness, include the employment of a random sampling approach for the physical responses, as well as the inclusion of a large number of participants in the study.

## Conclusions

Findings from this study have shown that participants who were above 60 years of age were more likely to accept a vaccine. Therefore, public enlightenment for COVID-19 vaccine uptake can be strategically targeted at younger populations, who evidence suggests may have a less favourable disposition towards being vaccinated against the disease.

As further revealed by this study, more than a third of the study participants expressed authority issues with respect to COVID-19 vaccination. Based on these findings, identifying and involving cultural and religious leaders, who citizens trust, in public enlightenment campaigns may help promote vaccine acceptance.

Whilst this study provides evidence of COVID-19 vaccine hesitancy in Nigeria, it also revealed new insights relating to attitudes associated with the public health intervention. This emergent body of evidence can form the basis for the development of contextual strategies to address vaccine hesitancy, as well as support immediate policies and guidelines in Nigeria and other similar settings.

## Supporting information

S1 File Questionnaire. (DOCX)
