# Supplementary material for: Attitudes towards COVID-19 vaccination: A cross sectional study in the Federal Capital Territory, Nigeria
Source: PLOS Glob Public Health. 2024 Apr 18;4(4):e0002589. doi: 10.1371/journal.pgph.0002589 (PMC11025807; doi:10.1371/journal.pgph.0002589)
Supplement: S1 File — (DOCX) [file pgph.0002589.s001.docx]

**NATIONAL INSTITUTE FOR PHARMACEUTICAL RESEARCH AND DEVELOPMENT (NIPRD) IDU, ABUJA.**

**Attitude towards COVID-19 vaccination in Nigeria**

**Introduction:**

Vaccine hesitancy was identified by the World Health Organization as one of the top ten global health threats. There is an unprecedented effort to make COVID-19 vaccine available globally, much is however not known about its acceptance in Nigeria and other Africa countries. This study therefore aimed at assessing the acceptance of COVID-19 vaccine in Nigeria. Please fill the questionnaire by ticking (√) the most appropriate option(s). Your views or options will be treated confidentially.

**Section A: Demography**

1. **Gender**

| Male |  | Female |  |
| --- | --- | --- | --- |

1. **Age**

| 18-30 |  | 31-40 |  | 41-50 |  | 51-60 |  | Above 60 |  |
| --- | --- | --- | --- | --- | --- | --- | --- | --- | --- |

1. **Highest Educational Level**

| Primary School |  | Secondary School |  | National Diploma/NCE |  | First Degree/HND |  | Postgraduate Level |  |
| --- | --- | --- | --- | --- | --- | --- | --- | --- | --- |

1. **Occupation**

| Unemployed |  | Self-employed |  | Private sector |  | Government sector |  | Retired |  | Others, please specify ………………………………. |
| --- | --- | --- | --- | --- | --- | --- | --- | --- | --- | --- |

1. **Profession**

| Healthcare |  | Non-Healthcare |  |
| --- | --- | --- | --- |

**Section B: Attitude towards COVID-19 Vaccine**

|  | **Statement** | **Strongly Agree** | **Somewhat Agree** | **Somewhat Disagree** | **Strongly Disagree** |
| --- | --- | --- | --- | --- | --- |
|  | To what extent do you agree or disagree with each of the following: |  |  |  |  |
| 1 | The chance of getting COVID-19 is so low that a vaccine is not necessary. |  |  |  |  |
| 2 | I would accept to be vaccinated for COVID-19 |  |  |  |  |
| 3 | The government is making decisions in your best interest with respect to vaccination. |  |  |  |  |
| 4 | COVID-19 vaccination should be made compulsory for every citizens. |  |  |  |  |
| 5 | Pharmaceutical companies have your best health interest at heart for developing COVID-19 vaccine. |  |  |  |  |
| 6 | Non-Governmental Entities supporting COVID-19 vaccine research have your best interest at heart. |  |  |  |  |

1. Will you accept COVID-19 vaccine if your employer insists you must be vaccinated before getting employed?
2. Yes
3. No
4. Ever been infected with COVID-19?
5. Yes
6. No

**Thank you for taking your time to complete this questionnaire**
